# MS‐based strategies for identification of protein SUMOylation modification

**DOI:** 10.1002/elps.201900100

**Published:** 2019-06-27

**Authors:** Zenghua Sheng, Xixi Wang, Yanni Ma, Dan Zhang, Yanfang Yang, Peng Zhang, Hongxia Zhu, Ningzhi Xu, Shufang Liang

**Affiliations:** ^1^ State Key Laboratory of Biotherapy and Cancer Center West China Hospital Collaborative Innovation Center for Biotherapy Sichuan University Chengdu P. R. China; ^2^ Department of Urinary Surgery West China Hospital Sichuan University Chengdu Sichuan P. R. China; ^3^ Laboratory of Cell and Molecular Biology & State Key Laboratory of Molecular Oncology Cancer Institute & Cancer Hospital Chinese Academy of Medical Sciences Beijing P. R. China

**Keywords:** MS, Peptide enrichment, Small ubiquitin‐like modifiers (SUMOs), SUMOylation, Ubiquitylation

## Abstract

Protein SUMOylation modification conjugated with small ubiquitin‐like modifiers (SUMOs) is one kind of PTMs, which exerts comprehensive roles in cellular functions, including gene expression regulation, DNA repair, intracellular transport, stress responses, and tumorigenesis. With the development of the peptide enrichment approaches and MS technology, more than 6000 SUMOylated proteins and about 40 000 SUMO acceptor sites have been identified. In this review, we summarize several popular approaches that have been developed for the identification of SUMOylated proteins in human cells, and further compare their technical advantages and disadvantages. And we also introduce identification approaches of target proteins which are co‐modified by both SUMOylation and ubiquitylation. We highlight the emerging trends in the SUMOylation field as well. Especially, the advent of the clustered regularly interspaced short palindromic repeats/ Cas9 technique will facilitate the development of MS for SUMOylation identification.

**Abbreviations**
AA: amino acids
CRISPR: clustered regularly interspaced short palindromic repeats
HDR: homology‐directed repair
His: histidine
IP: immunoprecipitation
K: lysine
N: asparagine
PRISM: protease‐reliant identification of SUMO modification
PTMs: post‐translational modifications
R: arginine
SENPs: SUMO‐specific proteases
SUMO: small ubiquitin‐like modifier
T: threonine
V: valine
WALP: wild‐type α‐lytic protease

## 1 Introduction

SUMOylation is one highly conserved and widely existing protein post‐translational modification (PTM) in various critical biological processes, including gene expression regulation, DNA damage repair, intracellular transport, pre‐mRNA splicing, and protein degradation 1, 2, 3, 4, 5. The small ubiquitin‐like modifier (SUMO) family contains four SUMO paralogs which are named SUMO‐1, SUMO‐2, SUMO‐3, and SUMO‐4 in mammalian cells 6. The SUMO‐2 and SUMO‐3 share 96% identity, whereas SUMO‐1 with 11.6‐kDa molecular weight shares 45% sequence identity with SUMO‐2 and SUMO‐3. SUMO‐4 is another member of the SUMO family, which has been studied relatively little.

All SUMOs are conjugated to a target protein by a same set of enzymatic biochemical reactions comprising the involvement of a heterodimeric SUMO activating enzyme E1, a single SUMO‐conjugating enzyme E2, and a SUMO ligase E3 7. Finally the free SUMO molecule, which is derived from SUMO‐specific proteases (SENPs)‐mediated deSUMOylation, is recycled to involve in another round of protein conjugation. SUMO interacts with the substrate proteins which possess the ε‐amino group of certain lysine (K) residues. The SUMO‐modified K residues often reside in the consensus motif composed of ψKxE or ψKxD (“ψ” represents a hydrophobic residue and “x” means any sort of amino acid residue) 8 or inverted consensus motif 9. Of course, the SUMO‐modified sites of non‐consensus K residues have been also reported 10.

With the technology development of peptide enrichment approaches and MS, more than 6000 SUMOylated proteins and about 40 000 SUMO acceptor sites have been identified 11, including transcription factors, nuclear proteins 12, especially those bindings located in the chromatin 13, and nuclear bodies 14. Nevertheless, the growing numbers of non‐nuclear SUMO‐modified proteins have been also reported 15.

Both SUMOylation and deSUMOylation are highly dynamic and well‐balanced in normal cellular activities. SUMOylation is essential for maintenance of genome integrity and regulation of intracellular signaling. Abnormal SUMOylation is relative to multiple diseases, including bacterial infections, diabetes, cleft lips, and cancers 16, 17. To understand the functional behavior of SUMOylation between health and disease, it is pivotal to determine whether or how SUMOylation takes place in a protein and which residues are SUMOylated. When SUMO is attached in a modified protein, mapping the exact K residue is a critical step to get further insight into the function of SUMOylation. The identification of SUMO‐modified sites in protein substrates by MS is challenging and developing rapidly 18.

In this review, we summarize several popular approaches that have been developed for the identification of SUMOylated proteins in human cells, and further compare their technical advantages and disadvantages. And we also introduce identification approaches of target proteins which are co‐modified by both SUMOylation and ubiquitylation. At last, we highlight the emerging trends in this field. Moreover, the advent of the clustered regularly interspaced short palindromic repeats (CRISPR)/Cas9 technique will facilitate MS identification for SUMO‐modified proteins.

## 2 MS identification of SUMO modifications

It is pivotal to identify SUMO‐modified substrates and SUMO acceptor sites at cell endogenous level for understanding SUMOylation‐involved biological processes. MS is a leading technology for investigating cellular proteomics, and PTMs 6, 19, 20, 21, 22, 23. Over 200 types of PTMs have been reported, and at least 8 different modification forms have been exactly identified by MS, including acetylation, glycosylation, ubiquitylation, methylation, phosphorylation on serine and threonine (T), adenosine diphosphate ribosylation, and proline isomerization and so on 21, 22, 23. However, MS identification of endogenous SUMOylated proteins remains challenging due to several aspects. Firstly, the abundance of SUMO‐modified proteins is very low in vivo, while the deSUMOylation protease activity of SENP is relatively high in cell lysates 24, which leads to SUMO conjugation lost rapidly in the absence of SUMO inhibitors. In addition, SUMO leaves a larger peptide signature after trypsin digestion, which produces complex MS fragmentation patterns. Considering these obstacles in MS identification, an ectopically mutant SUMO tag is usually introduced to express in cells for following enrichment and identification of SUMO‐modified proteins. So, we roughly classify these methods into the mutant SUMOs and non‐mutant SUMOs techniques.

### 2.1 Mutant SUMOs tagging

#### 2.1.1 SUMO‐tagging peptide characterization

**Figure 1:**
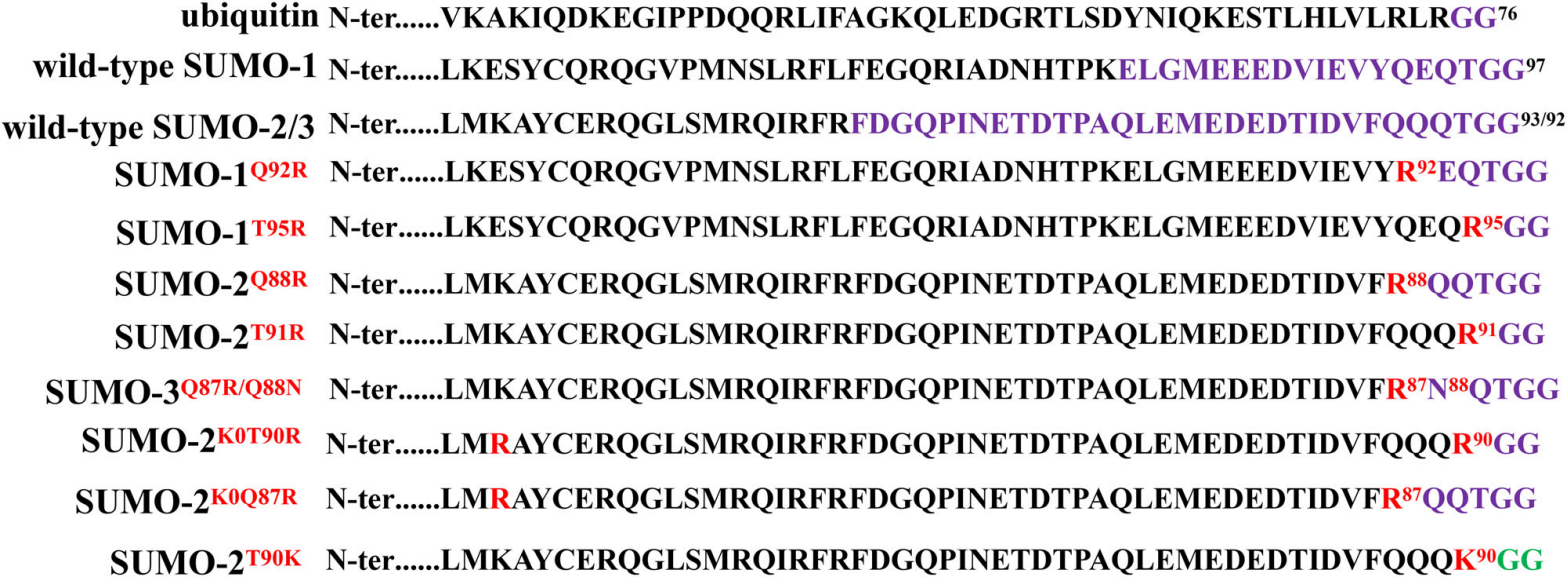
Schematic diagram of SUMO variants. Comparison of C‐terminal sequences in mature ubiquitin, wild‐type SUMO‐1, wild‐type SUMO‐2/3, SUMO‐1^Q92R^ (Q92 mutation to R), SUMO‐1^T95R^ (T95 mutation to R), SUMO‐2^Q88R^ (Q88 mutation to R), SUMO‐2^T91R^ (T91 mutation to R), SUMO‐3^Q87R/Q88N^ (Q87 and Q88 double mutation to R and N respectively), SUMO‐2^K0T90R^ (all K replaced with R, meanwhile an additional R is introduced at the AA90), SUMO‐2^K0Q87R^ (all K in replaced with R, meanwhile a R is introduced at AA87), SUMO‐2^T90K^ (T90 mutation to K). The length of the tryptic remnant is indicated by the first trypsin‐cleavable residue, and the peptide remnants are highlighted in purple. Similarly, the length of the Lys‐C remnant is indicated by the first Lys‐C‐cleavable residue, and the peptide remnants are highlighted in green. And the introduction of the mutation site is highlighted in red.

Trypsin belongs to a serine protease, which cleaves C‐terminal of K and arginine (R) residues. By tryptic digestion, the endogenous SUMO‐1 or SUMO‐2/3 remnant left on the peptides is made up of 19 and 32 amino acids (AA) respectively, whereas the ubiquitin remnant is composed of only two AA (Fig. 1). However, the tryptic peptides, more than 3 kDa in size, will greatly hamper the resolution effects of the SUMO‐modified peptides due to the current sensitivity of MS identification. In addition, their variable stoichiometry can complicate the interpretation of the corresponding product ion spectra 25.

Currently an ectopic expressing mutant SUMO tag fused with a target protein is the most common approach to solve the SUMO‐tagging peptide fragment length by tryptic digestion. For example, the R residue is introduced at the AA 92 site (Q mutated to R, SUMO‐1^Q92R^) 26, 27 or 95 (T mutated to R, SUMO‐1^T95R^) of SUMO‐1 28 to shorten the SUMO1‐tagging peptide generated after tryptic digestion. Overall, the SUMO‐1^Q92R^ variant contains a “EQTGG” tag by tryptic digestion, and SUMO‐1^T95R^ variant generates a KGG tag (Fig. 1).

Similarly, R residue is introduced at positions 88 (SUMO‐2^Q88R^) 26, 27, 91 (SUMO‐2^T91R^) 28 or 90 (SUMO‐2^K0T90R^) 9 of the SUMO‐2 tag. Through tryptic digestion, the SUMO‐2^Q88R^ variant contains a “QQTGG” tag, and the SUMO‐2^T91R^ and SUMO‐2^K0T90R^ variants will generate a KGG tag (Fig. 1). Moreover, a double‐sites mutant SUMO‐3 plasmid vector pHis_6_‐SUMO‐3^Q87R/Q88N^ is developed to introduce into cells, by which the variant SUMO‐3 generates a NQTGG tag through tryptic digestion (Fig. 1) 26, 27. Compared with the QQTGG remnant, the NQTGG has an important advantage that the asparagine (N) residue does not cyclize on the N‐terminus of the remnant, which improves the peptide MS identification. Besides, several K‐deficient multiple‐mutation SUMO tags had been reported, including the two similar SUMO‐2 variants SUMO‐2^K0T90R^ 9 and SUMO‐2^K0Q87R^ 29. The typical characteristic of K‐deficient SUMO mutants is that all K residues in the SUMO tag are replaced by R residues. For example, in the multiple‐mutation plasmids pSUMO‐2^K0T90R^ 9 and pSUMO‐2^K0Q87R^ 29, all K are replaced with R in the SUMO‐2, meanwhile R residue is also introduced at position 90 (T90R) or 87 (Q87R) (Fig. 1). These SUMO‐2 variants behave very similar to the wild‐type SUMO‐2, except for SUMO polymerization. For the variant SUMO‐2^K0T90R^ or SUMO‐2^K0Q87R^, a KGG or QQTGG tag will be produced by tryptic digestion, which greatly shortens the SUMO‐2‐branched peptide and contributes to MS identification.

The trypsin and endoproteinase Lys‐C are the most commonly selected digestion enzymes to cleave the mutant SUMO‐modified proteins to obtain specific patterned peptides. The endoproteinase Lys‐C is a protease, which specifically cleaves C‐terminal of K (except when connected with R) and produces larger peptides than trypsin. Therefore, Tammsalu et al introduced a K residue at the 90‐AA position of the SUMO‐2 protein (SUMO‐2^T90K^) to confer an action site of endoproteinase Lys‐C 30. When SUMO‐2^T90K^ is digested with Lys‐C, a KGG tag generates to avoid the false‐positive identification of ubiquitylation sites by MS (Fig. 1), which differs from the cleavage patterns of the variant SUMO‐1^T95R^ and SUMO‐2^T91R^ with trypsin.

#### 2.1.2 Enrichment of the SUMO‐modified proteins and peptides

An efficient enrichment of the low‐abundance of SUMO‐modified proteins or peptides from a variety of cellular proteins is necessary for the next MS identification. Several affinity tags are applied for the target protein purification, including histidine (His) 9, 26, 27, 28, 29, 30, hemagglutinin 31, and Flag tags 32. The His tag is the most used one for SUMO labeling, because its tagging with a target protein still retains the property and the substrate specificity of the native SUMO modification. Even under highly denaturing conditions, the His‐tagging SUMO‐conjugated proteins still will be enriched by nickel chromatography, while the co‐combined factors will be removed to decrease interference.

**Figure 2:**
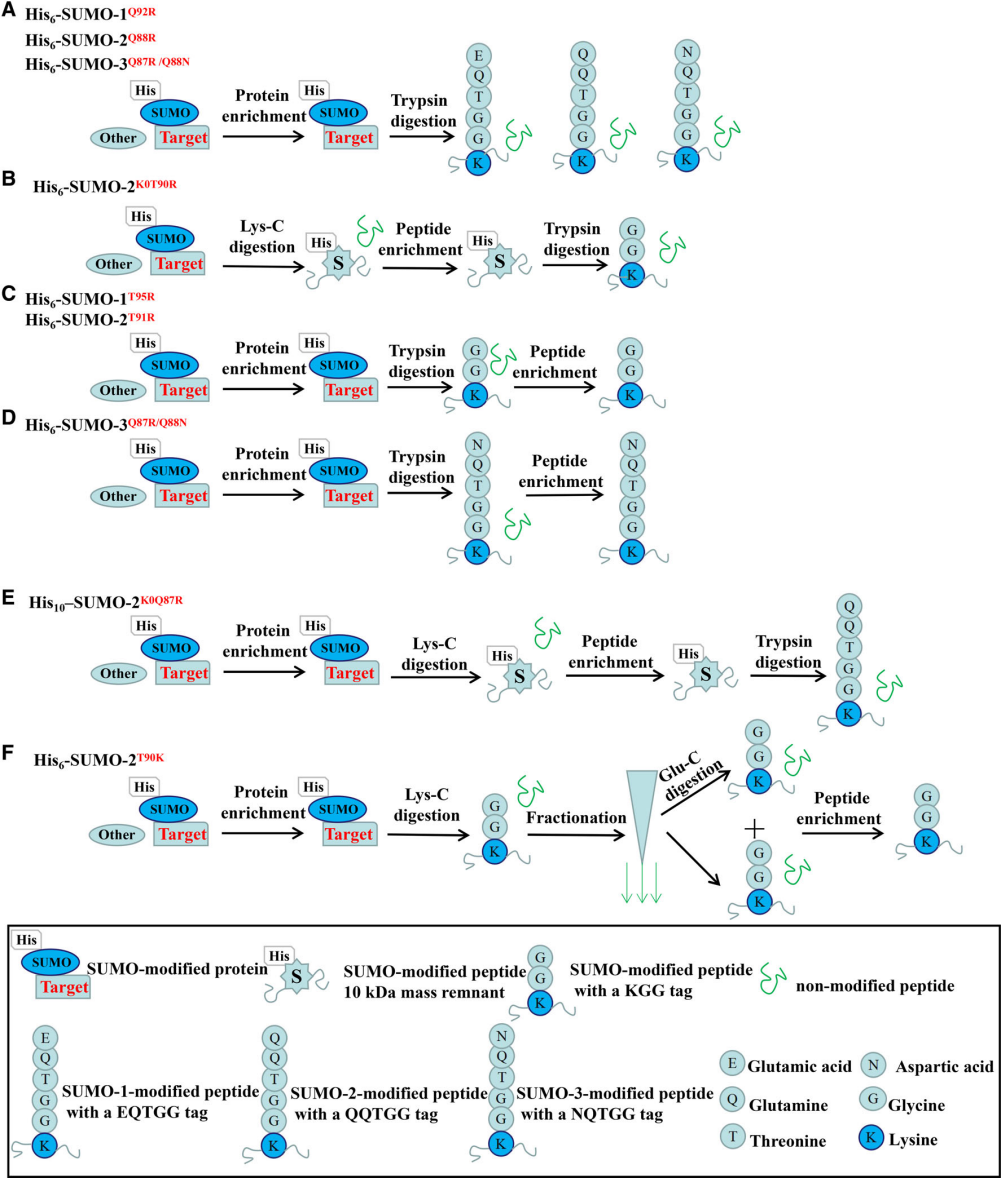
The mutant SUMOs system for enrichment of the SUMO‐modified proteins and peptides. (A) The identification strategy for SUMO‐modified sites based on the His_6_‐SUMO‐1^Q92R^, His_6_‐SUMO‐2^Q88R^ orHis_6_‐SUMO‐3^Q87R/Q88N^ mutants with one‐step enrichment and one‐step digestion. Proteins conjugated to the SUMO mutants are enriched by pulling‐down via His tag, and digested with trypsin. (B) The SUMOylation identification based on the His_6_‐SUMO‐2^K0T90R^ mutant with one‐step enrichment and two‐step digestion. Proteins conjugated to His_6_‐SUMO‐2^K0T90R^ are digested with Lys‐C, enriched by immobilized metal affinity chromatography, and re‐digested with trypsin. (C) The SUMO‐modified sites are identified using the His_6_‐SUMO‐1^T95R^ or His_6_‐SUMO‐2^T91R^ mutants with two‐step enrichment and one‐step digestion. Proteins conjugated to His_6_‐SUMO‐1^T95R^ or His_6_‐SUMO‐2^T91R^ are caught by pulling‐down of His tag, digested with trypsin, re‐concentrated by co‐IP using the anti‐K‐ε‐GG antibody. (D) The identification of SUMO‐modified sites using the His_6_‐SUMO‐3^Q87R/Q88N^ mutant with two‐step enrichment and one‐step digestion. Proteins conjugated to His_6_‐SUMO‐3^Q87R/Q88N^ are enriched by pulling‐down of His tag, digested with trypsin, concentrated by co‐IP using the anti‐NQTGG antibody. (E) The SUMO‐modified sites are identified based on the mutant His_10_–SUMO‐2^K0Q87R^ with two‐step enrichment and two‐step digestion. Proteins conjugated to His_10_–SUMO‐2^K0Q87R^ are pulled down by His tag, digested with Lys‐C, enriched by immobilized metal affinity chromatography, and re‐digested with trypsin. (F) The SUMO‐modified sites are identified based on the His_6_‐SUMO‐2^T90K^ mutant with two‐step enrichment and two‐step digestion. Proteins conjugated to His_6_‐SUMO‐2^T90K^ are pulled down by His tag, digested with Lys‐C, fractionated on StageTip, re‐digested with Glu‐C, and concentrated by co‐IP using the anti‐K‐ε‐GG antibody.

Generally, a one‐step or two‐step purification approach is applicable for enrichment of the mutant SUMOs‐tagging proteins. In one‐step purification of the target protein, the commonly essential step is affinity purification by the immobilized metal affinity chromatography via Ni^2+^ column 33. For example, the potential target SUMOylated proteins are enriched on Ni^2+^ column by one step His‐tagging purification using each of three mutants His_6_‐SUMO‐1^Q92R^, His_6_‐SUMO‐2^Q88R^, and His_6_‐SUMO‐3^Q87R/Q88N^ 26. And the enriched proteins are subsequently digested with trypsin for MS analysis (Fig. 2A) 26. By this approach, 17 precise SUMO‐modified sites were identified from 12 SUMO protein conjugates, including three new sites (K‐380, K‐400, and K‐497) on the protein promyelocytic leukemia by As_2_O_3_ treatment 26. Interestingly, two of these sites (K‐380 and K‐400) were shown previously to be ubiquitylated in vitro. So, determining the functions of these new SUMOylation sites on promyelocytic leukemia is helpful for further understanding the molecular mechanism of leukemia. After one‐step protein enrichment, two different digestion enzymes are usually combined to cleave the SUMO‐conjugated protein, which allows peptide to be cut smaller to improve peptide identification coverage. For instance, the His_6_‐SUMO‐2^K0T90R^‐tagging proteins, in which internal K are replaced by R, are experienced two‐enzyme digestion, including first Lys‐C and secondary trypsin digestion before MS analysis (Fig. 2B) 9. As the K‐deficient SUMO mutants are not sensitive to the digestion by Lys‐C, protein digestion by trypsin cleaves within the epitope of SUMO‐2/3, whereas digestion with Lys‐C will leave the epitope intact. Thus, after digestion with Lys‐C, the SUMO‐modified peptide fragment still carries a His‐label, which allows peptides to be enriched again on the nickel column. Subsequently, digestion with trypsin is performed to remove a large part of SUMO from the substrate peptides, leaving a short KGG tag. With this strategy, 103 SUMO‐2‐modified sites were identified in the endogenous target proteins 9. However, due to the use of trypsin for secondary digestion, sometimes the generated KGG tag leads to false‐positive identification of ubiquitylation sites.

In two‐step purification strategy, the first step is to enrich proteins, and the second step is to concentrate the digested peptides. When a SUMO‐modified protein is digested by trypsin, the number of non SUMO‐modified peptides is much more than that of SUMO‐modified peptides, which greatly reduces the sensitivity and accuracy of MS identification. Therefore, the target peptide enrichment is a crucial step before the MS identification. For instance, the two mutants His_6_‐SUMO‐1^T95R^ and His_6_‐SUMO‐2^T91R^ (Fig. 2C) 28 both have a special peptide pattern with a KGG tag after trypsin digestion. So, the specific KGG‐tagging peptides are efficiently enriched by immunoprecipitation (IP) using anti‐K‐ε‐GG antibodies, which improve MS identification for SUMO modification. A large number of SUMOylation sites had been discovered by combining stable isotope labeling of cell‐based quantitative proteomics and immunocapturing of SUMO‐modified peptides, including 295 SUMO‐1 and 167 SUMO‐2 acceptor sites on endogenous substrates of HeLa cells 28. However, this strategy still does not completely avoid the interference of false‐positive ubiquitylation sites. So far, based on the His_6_‐SUMO‐3^Q87R/Q88N^ system proposed by Galisson 26, Lamoliatte and his colleagues designed a specific peptide F{(NQTGG)K}GEC to immune rabbit, and a hybridoma cell line UMO 1‐7‐7 was screened for achieving monoclonal antibody, which recognizes the NQTGG‐tagging SUMO remnant peptides on modified K residues after the first step of protein digestion (Fig. 2D) [27]. Through the secondary peptide enrichment for the specific NQTGG sequences, totally 954 SUMO‐3‐modified sites in HEK293 cells were confirmed by quantitative proteomics 27. This strategy not only achieves the target peptides, but also effectively avoids the interference of false‐positive ubiquitylation sites.

In addition, the His_10_‐SUMO‐2^K0Q87R^ mutant is relatively simple and effective for the two‐step enrichment and two‐step digestion (Fig. 2E) 29. The His_10_ tag enables a single round of purification with a high yield and purity in contrast to the His_6_ tag commonly used in the field. In the His_10_‐SUMO‐2^K0Q87R^ approach, the first procedure of Lys‐C digestion is prepared for the next enrichment step for His_10_‐SUMO‐tagging peptides, and the secondary trypsin digestion is performed to obtain peptides containing a specific QQTGG tag for SUMO identification. A total of over 4300 SUMO‐modified sites in over 1600 proteins had been identified in human cells by this double enrichment /digestion method 29. Similarly, over 1000 SUMO‐modified sites were confirmed by a method with two‐step enrichment and two‐step digestion using a His_6_‐SUMO‐2^T90K^ variant (Fig. 2F) 30. As the endoproteinase Glu‐C has a high specific cleavage for peptide bonds which C‐terminal to glutamic or aspartic acids. So, the combination of endoproteinase Lys‐C and Glu‐C is opted to digest SUMOylated proteins. In this strategy, those specific KGG‐tagging peptides are produced by the first endoproteinase Lys‐C cleavage, and smaller peptides will be obtained by the secondary Glu‐C digestion 30.

### 2.2 Non‐mutant SUMOs techniques

Although the mutant SUMO approach is effective in mapping SUMO‐modified sites, there are still some limitations as following. One of the most typical drawbacks is unable to discriminate between cell endogenous SUMOylation and the artificial SUMO‐modified substrate residues induced by introduce of extra exogenous mutant SUMO tag. As a slight overexpression of SUMO or the presence of mutant sequences will cause attachment of unnatural SUMOs to K sites of proteins. Besides, these methods all require exogenous expression of mutant version of SUMO, which precludes analysis of SUMO‐modification sites in native settings or from animal tissues and clinical samples. So the non‐mutant SUMOs tagging system is also urgent to improve for SUMOylation identification.

**Figure 3:**
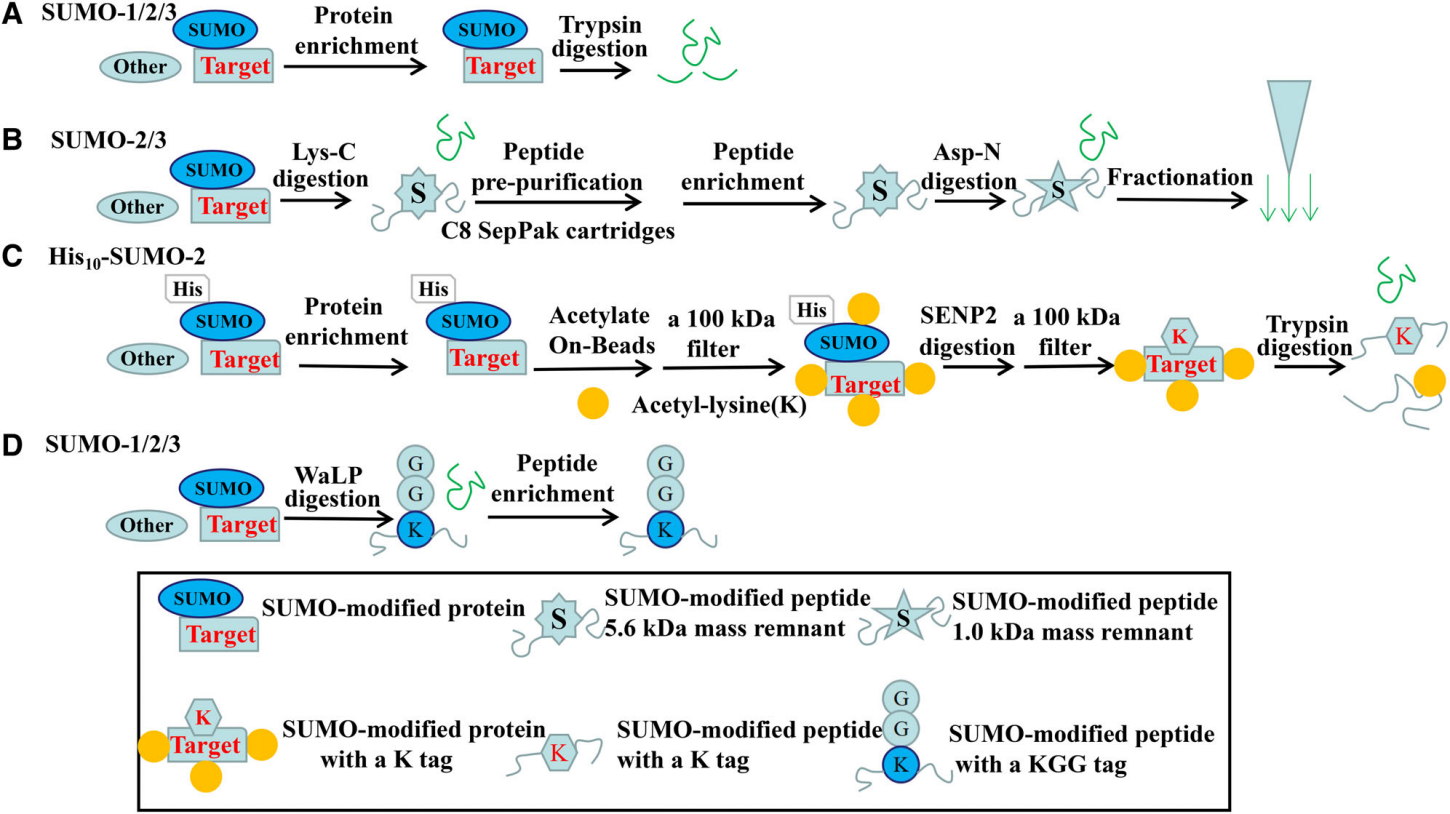
The non‐mutant SUMOs for enrichment of the SUMO‐modified proteins and peptides. (A) A strategy for identifying endogenous SUMO‐1/2/3 sites. Proteins conjugated to SUMO are enriched by co‐IP using the SUMO antibody, digested with trypsin. (B) The double enzyme digestion by Lys‐C/Asp‐N for identifying endogenous SUMO‐2/3 sites. Proteins conjugated to SUMO are digested with Lys‐C, pre‐purified using C8 SepPak cartridges, enriched by co‐IP using the SUMO‐2/3 antibody, subjected to a second round of Asp‐N digestion, and fractionated on StageTip for following MS identification. (C) The PRISM method for identifying SUMO sites. Proteins conjugated to His_10_‐SUMO‐2 are enriched by pulling‐down via His tag, acetylated on‐beads using sulfosuccinimidyl‐acetate, fractionated by a 100 kDa filter, digested with SENP2 to generate a K tag, re‐fractionated by a 100 kDa filter, and re‐digested with trypsin. (D) The WaLP enzyme digestion for identifying endogenous SUMO‐1/2/3 sites. Proteins conjugated to SUMO are digested with WaLP, enriched by co‐IP using the anti‐K‐ε‐GG antibody.

A relatively simple protocol with one‐step enrichment and digestion is designed to identify SUMO‐modified sites via a wild‐type SUMO tag in mammalian cells 34. The SUMO‐modified proteins are enriched by IP using two well‐known monoclonal anti‐SUMO‐1 and anti‐SUMO‐2/3 antibodies. To reduce contaminations in IP, the minimal epitope‐spanning antibody peptides are used for selective elution of antigens under efficient elution conditions. The trypsin is still used to digest the SUMO‐binding partners after one‐step protein purification (Fig. 3A). More than 1000 proteins were identified in the SUMO‐associating immunoprecipitated complex, including many non‐specific SUMO‐binding proteins 34. By highly stringent selection criteria for data analysis, finally 232 endogenous SUMO target candidates were confirmed with high credibility 34. However, a huge flaw in this strategy is that the tryptic remnant exceeds 3 kDa in size. Subsequently, an improved approach with two‐step enrichment and two‐step digestion 35 has been developed to overcome the disadvantage of Janina Becker's solutions 34. The most critical procedure improvement is that substitution of trypsin with endoproteinase Lys‐C to digest SUMO‐2/3 conjugates, which makes it more efficient to enrich the digested peptides. In order to generate smaller peptides, the enriched SUMO‐modified peptides are subjected to a second round of digestion using Asp‐N enzyme, after which peptides are fractionated on a StageTip apparatus (Fig. 3B). So far, it is the largest reported number that 14 869 endogenous SUMO‐2/3 acceptor sites were identified in human cells with heat stress and proteasomal inhibition 35.

Another problem of non‐mutant SUMOs techniques is great difficulty in using the Mascot database search engine to match the non‐mutant SUMO‐containing peptides which are digested by trypsin or Lys‐C without special peptide patterns. To solve this bottleneck, Hendriks and colleagues developed a protease‐reliant identification of SUMO modification (PRISM) method 36, which includes one step enrichment and two steps digestion. The key improvement of PRISM is the introduction of specific peptide patterns with a K tag after the SENP2 cleavage of target proteins. In order to enable to produce an exposed K residue only in the modified site, the enriched proteins are acetylated on‐beads by sulfosuccinimidyl‐acetate under highly denaturing conditions, which enables efficient blocking of K residue for avoiding next SENP2 cleavage (Fig. 3C). In the method procedures, the buffer conditions are sufficiently strict, which is compatible with the next procedures, including chemical labeling of all K sites, function of recombinant SUMO protease and function of trypsin. This PRISM technique is feasible to catch cell endogenous SUMOylated residues such as 200 dynamic SUMO‐modified sites in response to heat shock 36.

More excitingly, the wild‐type α‐lytic protease (WaLP) is efficiently introduced for the SUMOylation identification, which greatly widens MS identification of natural SUMO modifications from clinical tissues 37. As WaLP prefers to cleave after T residues and rarely cleaves after R. WaLP specifically cleaves at the C‐terminal TGG sequence of all SUMO paralogs, by which a SUMO‐remnant KGG tag is obtained at the position of SUMO attachment in a target protein 37. In addition, it generates peptides with the same average length as trypsin digestion despite its more relaxed substrate specificity. The general process of WaLP method includes one step enrichment and one step digestion. After WaLP digestion, peptides are directly enriched using the anti K‐ε‐GG specific antibody. Under completely native conditions, the SUMO‐modified sites from tissue samples are identified by simply substituting WaLP for trypsin (Fig. 3D). By WaLP method, 1209 SUMO‐modified sites are confirmed on endogenous substrates of human cells 37.

## 3 Comparisons of mutant and non‐mutant SUMOs methods

The high‐throughput MS methods have been widely applied to explore SUMO‐modified substrates and SUMO acceptor sites. The strong points and drawbacks are concomitant for the mutant and non‐mutant SUMOs approaches. Here, we make a comparison summary of several SUMOylation identification solutions (Table 1).

**Table 1:** Technique comparisons of mutant and non‐mutant SUMOs methods

SUMO types	Proteolytic enzyme	Unique enzymatic digestion patterns	Times of enrichment	Concentration of enriched target peptides	Peptide remnant under 3 kDa	Existing unnatural SUMO attachment	Available for tissue and clinical sample	False‐positive identification of ubiquitylation sites	Number of SUMO‐1 modified sites	Number of SUMO‐2/3 modified sites	Ref.
His_6_‐SUMO‐1^Q92R^ His_6_‐SUMO‐2^Q88R^ His_6_‐SUMO‐3^Q87R /Q88N^	Trypsin	√	One	Low	√	√	×	×	17		26
His_6_‐SUMO‐2^K0T90R^	Lys‐C/Trypsin	√	One	Low	√	√	×	√	×	103	9
His_6_‐SUMO‐1^T95R^ His_6_‐SUMO‐2^T91R^	Trypsin	√	Two	High	√	√	×	√	295	167	28
His_6_‐SUMO‐3^Q87R /Q88N^	Trypsin	√	Two	High	√	√	×	×	×	954	27
His_10_–SUMO‐2^K0Q87R^	Lys‐C/Trypsin	√	Two	Medium	√	√	×	×	×	4300	29
His_6_‐SUMO‐2^T90K^	Lys‐C/Glu‐C	√	Two	High	√	√	×	×	×	1000	30
SUMO‐1/2/3	Trypsin	×	One	Low	×	×	√	×	232		34
SUMO‐2/3	Lys‐C/Asp‐N	×	Two	Medium	√	×	√	×	×	14869	35
His_10_‐SUMO2	SENP2/Trypsin	√	One	Low	√	√	×	×	×	751	36
SUMO‐1/2/3	Walp	√	One	High	√	×	√	√	1209		37

In mutant SUMOs techniques, all of mutant SUMOs methods use a form of SUMO with one or more AA substitution at the C‐terminus, which confers to the SUMOylated peptides with an easily recognized pattern in MS. Another common feature is the usage of His tag for enhancing target protein abundance, purity, and overall efficiency. Thus, the site‐level proteomic approach avoids the common pitfall in protein‐level proteomics, while in the latter method some contaminant proteins are falsely identified as SUMO targets. To further catch the SUMO‐modified peptides for SUMOylation identification, a second purification for peptides is usually performed by recognizing the exposed specific‐tag residues, such as using the pHis_6_‐SUMO‐3^Q87R/Q88N^ 27, pHis_6_‐SUMO‐1^T95R^ 28, pHis_6_‐SUMO‐2^T91R^ 28, pHis_10_‐SUMO‐2^K0Q87R^ 29, and pHis_6_‐SUMO‐2^T90K^ plasmid transfection methods 30. The secondary purification method is largely owing to efficient purification of peptides instead of proteins. Sometimes, a secondary enzyme digestion is performed to obtain smaller peptides, such as in the His_6_‐SUMO‐2^K0T90R^ 9, His_10_‐SUMO‐2^K0Q87R^ 29, and His_6_‐SUMO‐2^T90K^ system 30.

However, there are still several common drawbacks for the mutant SUMO approaches: (1) it is possible that slight overexpression of SUMO or the presence of mutant sequences could cause unnatural SUMO attachment; and (2) the introduction of exogenous SUMO is limited to cell sample identification, which is not available for animal tissue samples and clinical tissue or fluid samples. Furthermore, the His_6_‐SUMO‐1^T95R^, His_6_‐SUMO‐2^T91R^, and His_6_‐SUMO‐2^K0T90R^ mutant systems probably have false‐positive identification of ubiquitylation sites, because both the mutant SUMO‐tagging protein and the ubiquitinated protein will produce a same KGG tag by trypsin digestion. As the cleavage site of Lys‐C is different from trypsin, then an ubiquitylated protein does not produce a KGG tag by Lys‐C cleavage in His_6_‐SUMO‐2^T90K^ method. In addition, the His_6_‐SUMO‐3^Q87R/Q88N^ and His_10_‐SUMO‐2^K0Q87R^ systems generate NQTGG and QQTGG tags by trypsin digestion, which can overcome interference of false‐positive ubiquitylation sites. However, compared with the wild QQTGG remnant, the NQTGG has an important advantage that the N residue does not cyclize on the N‐terminus of the remnant, which can improve the target peptide identification.

Compared to the mutant SUMOs strategies, the current non‐mutant SUMOs methods are more prone to reflect the real SUMOylation profile in organisms. However, the non‐mutant SUMOs methods still have some limitations, mainly including low efficiency of target peptide enrichment and the tryptic remnant more than 3 kDa in size 34. Latter Hendriks and colleagues described another superior system with a secondary digestion/enrichment 35. But, they all share common weakness without special peptide patterns which cannot easily recognized by MS after enzyme digestion.

The PRISM strategy has a special pattern with K tag, and the peptides identified by MS are also small. But, to generate the K tag, the design strategy is particularly demanding, which makes the experimental procedures too tedious. Due to the introduction of exogenous pHis_10_‐SUMO‐2 plasmid, this PRISM method is still not applicable for animal tissue samples and clinical tissue or fluid samples, and it could cause unnatural SUMO attachment. In contrast, the application of WALP digestive enzymes greatly promotes MS identification efficiency for SUMO modification. The WALP method belongs to non‐mutant SUMO technology, and it also produces a KGG tag to make peptide purification a reality. Meanwhile, WaLP generates peptides of the same average length as trypsin despite its more relaxed substrate specificity. However, the WALP has various shortcomings, including the inability to distinguish SUMO family members and high potential for false‐positive identification of ubiquitylation sites as SUMOylation. Besides, it is certain difficulties that the WALP enzyme needs to maintain high activity in cellular processing.

Considering the advantages and disadvantages of the above methods, we can design and apply these strategies in different combinations according to the specific experimental purposes. For example, a transcription factor of arabidopsis is customized for the study on plant SUMOylation sites by the aided of three plasmids 38. Based on this key issue, designing a convenient and efficient plasmid system pGEX‐6p‐3D is also a huge improvement. Overall, the joint application of different proteases is also a good way, like as the combination of endoproteinase Lys‐C and trypsin 39. In addition, it should be combined with classic biochemical experiments, such as verification of functions through AA point mutation, which is also a complementary method.

## 4 MS identification of co‐modified proteins by SUMOylation and ubiquitylation

A collaborative crosstalk also exists between SUMOylation and other PTMs. Besides SUMOs have extensively modified multiple enzymes, including ubiquitin ligase, ubiquitin protease, methyltransferase, demethylase, acetyltransferase, deacetylase, kinase, and phosphatase, to regulate their functions. For instance, the antiviral kinase activity of TANK‐binding kinase 1 is enhanced due to a SUMO modification at K694 40. The K residues of substrates are covalently bonded with SUMO and ubiquitin, and the biochemical process of modification is similar. SUMO alters protein ubiquitylation level in a synergistic or antagonistic manner. For example, the SUMO inhibitor 2‐D08 decreases SUMOylation at the chromosome axis, but it also downregulates both ubiquitylation and the proteasome, which implies that SUMO‐modified proteins become substrates of the ubiquitin‐proteasome pathway 41. Therefore, discovery of the co‐modification of SUMOylation and ubiquitylation is also of great significance for understanding crosstalk of several PTMs in physiology and diseases states.

**Figure 4:**
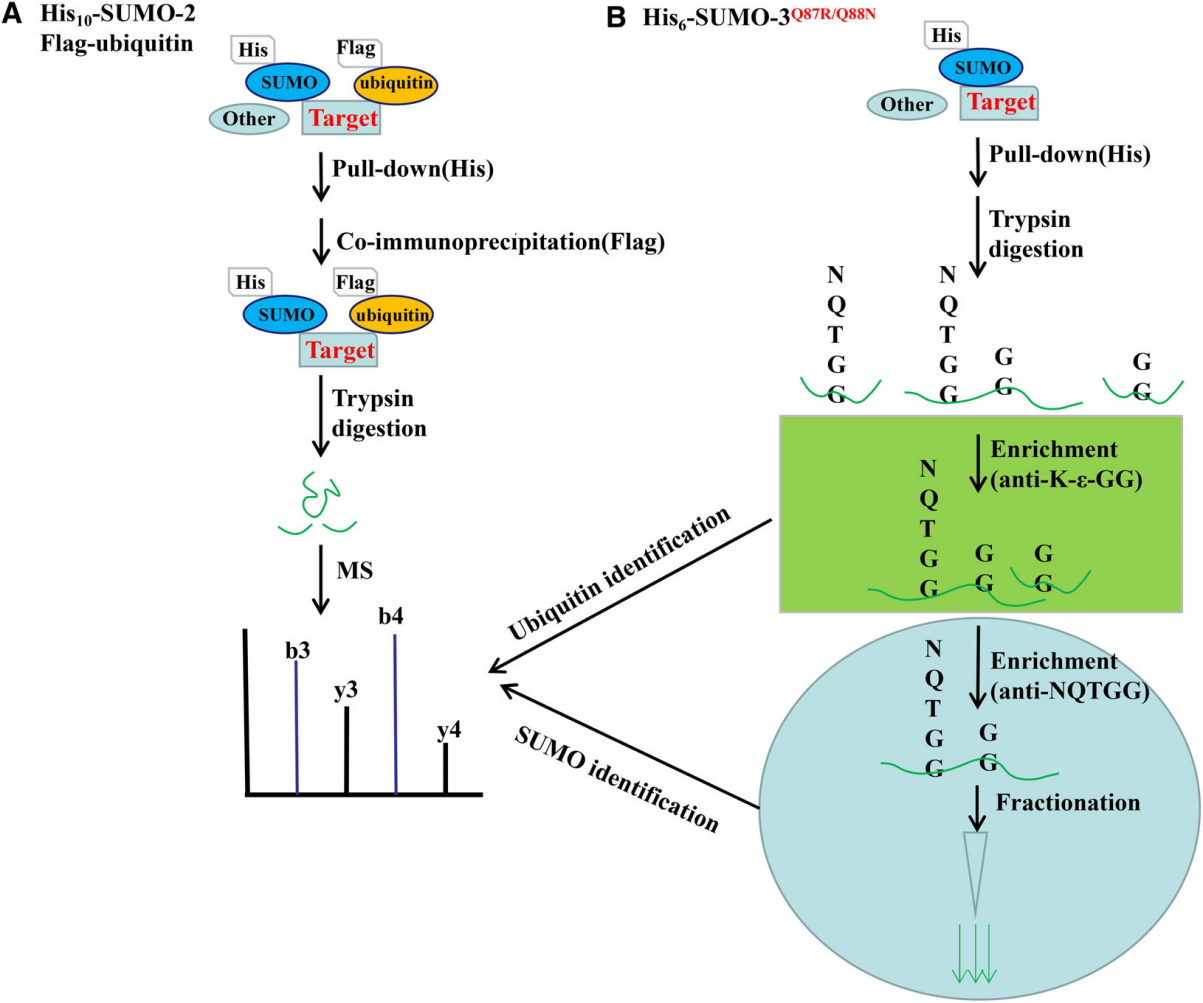
Identification of target proteins co‐modified by SUMO and ubiquitin is achieved using the His_10_‐tagged SUMO‐2/Flag‐tagged ubiquitin approach (A), and (B) His_6_‐SUMO‐3^Q87R/Q88N^ method.

So far it is challenging to figure out PTMs‐mediated signaling crosstalk on an unbiased proteome‐wide level. The pHis_10_‐SUMO‐2 and pFlag‐ubiquitin plasmids were steadily co‐expressed in cells to study the co‐modified target proteins, and a majority of co‐modified proteins by SUMO and ubiquitin are precisely identified 42. The main manipulation includes the SUMO‐2‐conjugated proteins caught by His_10_‐tagging pull‐down, removal of the free His_10_‐SUMO‐2, and following enrichment of co‐modified proteins by SUMO‐2 and ubiquitin through an IP against anti‐Flag antibody, finally peptides digested by trypsin to be analyzed by MS (Fig. 4A). Totally 498 proteins were confirmed to be significantly co‐modified by SUMOylation and ubiquitylation 42. Although the technique process is relatively simple, it is difficult to popularize the case‐by‐case performance.

Another attempt was applied through a double mutant plasmid pHis_6_‐SUMO‐3^Q87R/Q88N^ stably expressing in HEK293 cells to identify co‐modified proteins 43. The proteins were adsorbed by nickel column, digested directly on the beads using trypsin, and generated a NQTGG tag remnant on the SUMOylated peptides or a kGG remnant within the ubiquitinated peptides. The co‐modified peptides were concentrated by IP against the anti‐K(GG) and anti‐K(NQTGG) antibodies. It usually takes 3 days starting from cell pellet collection and yielding more than 8000 SUMO‐modified sites and 3500 ubiquitylation sites from 16 mg of cell extract 43. It is obviously superior to Willemstein's strategy 42, as this strategy adds enrichment steps of ubiquitylated peptides to achieve identification of SUMOylation and ubiquitin co‐modifications.

## 5 Perspectives

SUMOylation is conserved in all eukaryotes for the maintenance of genomic integrity 44. With the development of MS methodologies, more than 6000 proteins have been identified as the SUMOylation targets. Since SUMOylation is closely associated with the pathological processes, carcinogenesis and tumor metastasis, an efficient identification of SUMOylation is important to comprehensively understand roles of SUMOylated proteins in biological sciences and biomedicine. Furthermore, SUMOylation has cross‐talking with other PTMs, such as phosphorylation 45 and ubiquitylation 46, to regulate protein kinase activities and improve the stability of complex signaling pathways. Due to the low abundance and ultrasensitive regulation effect of SUMOylation, several SUMO inhibitors with high efficiency and low toxicity have been developed to try for cancer therapy 47, 48.

So, it is of great importance to develop efficient identification approaches for endogenous SUMOylated proteins. A perfect SUMOylation identification approach should include simple experimental procedures, a minimum sample throughput, and possessing the capability of sensitive quantification and multiplexed analysis. Great efforts have been put into the development of efficient approaches. However, the number of method still remains low for identifying endogenous SUMO‐modified sites on a global proteome scale. This may be ascribed to the low abundance of SUMO‐modified proteins in vivo, and the lack of naturally occurring protease sites in the C‐terminal tail of SUMOylated proteins. Because of various limiting factors, no perfect method can satisfy the above demands to date.

Efficient enrichment of endogenous SUMO‐modified proteins from a wide range of mammalian cells and tissues is the first step of identification by MS. So, the development of SUMO‐specific antibodies will be the mainstream trend for MS identification of SUMO‐modified proteins. It is also very necessary to develop a new protease that can accurately cut SUMO and make the SUMO‐modified proteins expose special patterns which can be easily recognized by MS. Furthermore, precise de novo peptide sequencing is hindered by poor coverage of b and/or y ion series, which is a common problem in SUMOylation identification by MS. Recently, a protein digestive enzyme Ac‐LysargiNase has been reported to provide a better coverage and stronger signal of b ions compared to tryptic peptides 49, it also can work with trypsin to create a complementary ion series. So, the application of Ac‐LysargiNase in SUMOylation identification is also a technical improvement.

Interestingly, it is noticeable the CRISPR/Cas9 technology is a powerful DNA editing tool for introduction of specific base mutations through homology‐directed repair in mammalian cells 50, 51, 52, 53, 54, 55. For instance, Paquet et al. used CRISPR/Cas9 technology to precisely mutate the single nucleotide (CAT mutation to CGT) of presenilin 1 gene, finally resulting in the introduction of valine (V) residue to substitute the methionine146 site of presenilin 1 protein (presenilin 1^M146V^) in human induced pluripotent stem cells 52. Similarly, a specific single nucleotide mutation (ACG mutation to AGG) of SUMO‐1 gene will be achieved by CRISPR/Cas9 technology, which introduces R residue to replace the T95 site of SUMO‐1 protein (SUMO‐1^T95R^) in cells, and ultimately the identification of endogenous SUMOylation can be achieved. Currently the enhanced CRISPR/Cas9 system has been improved to increases the homology‐directed repair accuracy and precision site mutations 53, 54, 55, 56, by which the mutant SUMO system is feasible to perform in cell endogenous states to capture SUMOylated peptides for MS identification. Moreover, the CRISPR technology has potential to assist MS in identifying SUMO‐modified sites not only at the cellular level, but also in animal tissues, which greatly improves efficiency compared with the above‐mentioned traditional mutant SUMOs systems.

In summary, with the development and progress of science and technology, we firmly believe that new methods will enable systematic and unbiased study of protein SUMOylation in the future. For example, The integration of single‐molecule detection with the quantum dots has distinct advantages of short analysis time and high sensitivity 57, and it may hold great potential for further application in simultaneous measurement of multiple low‐abundant SUMOylation.

*The authors have declared no conflict of interest*.
